# Serum Oligomeric α-Synuclein and p-tau181 in Progressive Supranuclear Palsy and Parkinson’s Disease

**DOI:** 10.3390/ijms25136882

**Published:** 2024-06-23

**Authors:** Costanza Maria Cristiani, Luana Scaramuzzino, Andrea Quattrone, Elvira Immacolata Parrotta, Giovanni Cuda, Aldo Quattrone

**Affiliations:** 1 Neuroscience Research Center, Department of Medical and Surgical Sciences, University “Magna Graecia”, 88100 Catanzaro, Italy; costanza.cristiani@unicz.it (C.M.C.);; 2 Institute of Molecular Biology, Department of Medical and Surgical Sciences, University “Magna Graecia”, 88100 Catanzaro, Italy; 3 Department of Clinical and Experimental Medicine, University “Magna Graecia”, 88100 Catanzaro, Italy

**Keywords:** Parkinson’s disease, progressive supranuclear palsy, oligomeric α-synuclein, p-tau181

## Abstract

Clinical differentiation of progressive supranuclear palsy (PSP) from Parkinson’s disease (PD) is challenging due to overlapping phenotypes and the late onset of specific atypical signs. Therefore, easily assessable diagnostic biomarkers are highly needed. Since PD is a synucleopathy while PSP is a tauopathy, here, we investigated the clinical usefulness of serum oligomeric-α-synuclein (o-α-synuclein) and 181Thr-phosphorylated tau (p-tau181), which are considered as the most important pathological protein forms in distinguishing between these two parkinsonisms. We assessed serum o-α-synuclein and p-tau181 by ELISA and SIMOA, respectively, in 27 PSP patients, 43 PD patients, and 39 healthy controls (HC). Moreover, we evaluated the correlation between serum biomarkers and biological and clinical features of these subjects. We did not find any difference in serum concentrations of p-tau181 and o-α-synuclein nor in the o-α-synuclein/p-tau181 ratio between groups. However, we observed that serum p-tau181 positively correlated with age in HC and PD, while serum o-α-synuclein correlated positively with disease severity in PD and negatively with age in PSP. Finally, the o-α-synuclein/p-tau181 ratio showed a negative correlation with age in PD.

## 1. Introduction

Parkinson’s disease (PD) and progressive supranuclear palsy (PSP) belong to parkinsonisms, a group of neurodegenerative disorders characterized by bradykinesia possibly combined with rest tremor and rigidity. Although PSP displays a more aggressive phenotype and additional symptoms such as early postural instability and vertical gaze abnormalities, these signs may occur later or never [1,2]. This makes clinical diagnosis at early stages challenging and misdiagnoses common [3]. However, an accurate diagnosis would allow better patient management as well as a correct stratification of patients in clinical trials.

Currently, clinical diagnosis can be confirmed only by post-mortem assessment of neuropathological hallmarks: PD is a synucleinopathy characterized by aggregates of α-synuclein found within neuronal soma (Lewy bodies), while PSP is a tauopathy showing the presence of tufted astrocytes and oligodendroglial coiled bodies composed of aggregated 4R isoforms of tau protein [4]. These histological findings have prompted investigations on the potential role of α-synuclein and tau as biomarkers for parkinsonisms, and their measurement in vivo in body fluids.

Total α-synuclein levels were found to be reduced in the cerebrospinal fluid (CSF) of PD patients compared to healthy controls [5,6,7,8], while measurements in plasma/serum provided more variable results [9,10,11]. Accordingly, the diagnostic accuracy of α-synuclein was overall low [7,8,9]. More promising results have been obtained by the quantification of oligomeric α-synuclein (o-α-synuclein), considered the pathological form determining protein aggregation [12]. o-α-synuclein concentration in CSF from PD patients was consistently higher, showing a good diagnostic accuracy in distinguishing PD patients from healthy subjects [13,14,15,16], but there is less evidence in serum or plasma [17,18], with a couple of studies showing a significant increase in PD patients [19,20] and others showing values comparable to or even lower than controls [11]. Conflicting results have also been obtained in differentiating PSP patients from controls by dosing total or tangle-forming 181Thr-phosphorylated tau (p-tau181) [4] in CSF or plasma [21,22,23,24,25,26,27,28,29].

Despite the distinct involvement of α-synuclein and tau in parkinsonisms, their potential role as biomarkers in distinguishing between PD and PSP patients has been scarcely investigated [13,22,23,24,25,26,27,30]. Moreover, most of the assessments were performed in CSF, which can only be collected by invasive procedures.

In this study, we aimed to investigate the clinical utility of serum o-α-synuclein and p-tau181 in differentiating between PSP and PD, to identify diagnostic markers that can be easily assessed in clinical practice.

## 2. Results

### 2.1. Differences between Groups

In total, 27 PSP patients, 43 PD patients, and 39 HCs were included, whose demographic, clinical, and serum data are summarized in Table 1. No differences in age and sex distribution among groups as well as in disease duration between patients were detected. Conversely, PSP patients showed higher MDS—Unified Parkinson’s Disease Rating Scale part III (MDS-UPDRS-III) and Hoehn and Yahr (HY) Staging Scale scores, in accordance with the more aggressive disease [2].

**Figure 1:**
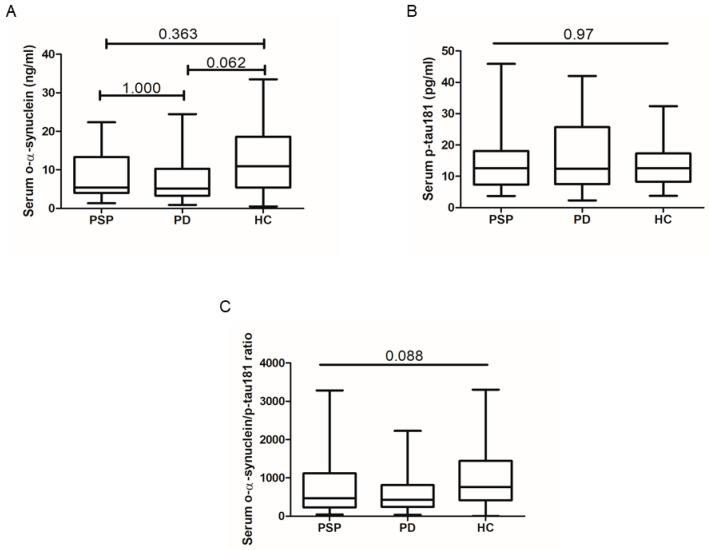
Serum concentration of o-α-synuclein (**A**) and p-tau181 (**B**) as well as the o-α-synuclein/p-tau181 ratio (**C**) in PSP patients (n = 27), PD patients (n = 43), and HCs (n = 39). Data are summarized as box plots, in which the lower, upper, and middle lines of the boxes represent the 25th percentile, 75th percentile, and median, respectively, while the limits of vertical lines indicate ranges. Shown *p*-values were obtained by a Kruskal–Wallis test followed by Bonferroni’s correction. o-α-synuclein = oligomeric α-synuclein; p-tau181 = 181Thr-phosphorylated tau; PSP = progressive supranuclear palsy; PD = Parkinson’s disease; HC = healthy control.

Regarding the selected biomarkers, only the serum concentration of o-α-synuclein showed a significant difference between groups, being slightly lower in PD patients compared to HCs, while serum p-tau181 levels were similar among groups. The o-α-synuclein/p-tau181 ratio was also not significantly different among groups. However, the significance of o-α-synuclein between PD and HC did not survive Bonferroni’s correction (Table 1 and Figure 1).

### 2.2. Correlation Analyses

Spearman’s correlation tests were carried out to assess the possible association of serum o-α-synuclein or p-tau181 concentration with demographic and clinical features in both PSP (Table 2) and PD (Table 3) patients. For the association between serum biomarkers and disease severity, the PSP Rating Scale and MDS-UPDRS-III were used for PSP and PD patients, respectively. Serum o-α-synuclein was shown to correlate negatively with age in PSP (Table 2) and positively associated with disease severity in PD (Table 3). Serum p-tau181 positively correlated with age in both PD and HC (Table 3 and Table 4).

## 3. Discussion

The current clinical diagnosis of PSP is mainly based on clinical signs, especially the presence of vertical supranuclear gaze palsy or slowness of vertical saccades [2]. However, such signs can occur never or late during the disease and may be challenging to evaluate [31]. Moreover, distinct PSP subtypes exist, with different severity and progression rates, which overall complicate the correct differentiation of PSP from PD, particularly at early stages [1,2]. Therefore, in recent years, intense research has been devoted to the identification of biomarkers to support clinical diagnosis.

Magnetic Resonance Imaging (MRI) of brain atrophy has provided useful measurements to correctly differentiate between PSP and PD patients, such as the midbrain-to-pons area ratio or the magnetic resonance parkinsonism index (MRPI and MRPI 2.0) [32,33]. Moreover, a simplified measurement, based on the ratio between the third ventricle width and the internal skull diameter, has recently been validated for differentiation of de novo PD from PSP patients [34]. Nevertheless, there is a growing interest in molecular biomarkers, potentially reflecting the underlying pathological alterations, which may be of great usefulness to support the clinical diagnosis especially if measurable in highly accessible fluids such as peripheral blood.

Since the main component of pathological inclusions observed in PD and PSP are α-synuclein and tau, respectively, these proteins have gained much attention as biomarkers, particularly in their pathological aggregation-prone forms (oligomeric α-synuclein and phosphorylated tau) [4]. Many studies have been conducted by dosing these proteins in CSF, regarded as the biological fluid that better recapitulates central nervous system (CNS) pathologies, showing that o-α-synuclein is a promising biomarker to distinguish PD patients from HCs, while results for p-tau181 are more conflicting [13,14,15,16,17,22,23,24,25]. However, lumbar puncture is an invasive procedure, while the measurement of these biomarkers in serum or plasma would be easier and have a higher compliance rate. Nevertheless, scarce literature is available regarding the capability of peripheral o-α-synuclein and p-tau181 to discriminate PSP and PD patients from controls or between parkinsonisms [17,18,26,27].

In this study, we demonstrated that serum o-α-synuclein and p-tau181 as well as their ratio did not distinguish PSP from PD patients. To our knowledge, this is the first study analyzing these biomarkers and their ratio using serum as a biological matrix to discriminate between these two diseases. Concerning serum o-α-synuclein in PD, we observed no significant difference between the PD group and controls, in contrast with data from CSF [13,25]. This lack of difference could be due to the presence in serum of o-α-synuclein of peripheral origin, for example, from the gastrointestinal tract [35] or erythrocytes [36], which could mask possible different amounts of o-α-synuclein derived from the CNS. Another discrepancy we observed compared to previous literature [17,18] is the tendency of PD patients to have lower levels of serum o-α-synuclein compared to controls. However, in one of these studies, a very small cohort was analyzed [17], while in the other study, o-α-synuclein quantification was not performed [18]. Moreover, both works employed in-house ELISA assays, while we used a commercial kit. Considering that synuclein oligomers can exist in several forms with different shapes and properties [12], it is challenging to compare results obtained by using different procedures and antibodies. The lower serum concentrations of o-α-synuclein we found in PD in comparison with healthy controls might reflect the enhanced aggregation in fibrils occurring in these patients, which would leave fewer free molecules to be detected. In addition, no difference was found between PD and PSP patients nor between PSP and control subjects. This is the first study investigating serum o-α-synuclein in these two different neurodegenerative diseases. A previous study in PD and PSP patients showed that CSF total α-synuclein levels were lower in PD patients than in patients with tauopathies such as PSP and Corticobasal Degeneration (CBD) [25]. With regard to p-tau181, our study confirms previous findings obtained in CSF [22,23,24,25] but not in plasma [27]. However, it has previously been demonstrated that these two matrices are not interchangeable with regard to p-tau181 measurements [37], which makes comparison difficult.

Collectively, our data indicate that serum o-α-synuclein and p-tau181 in serum do not mirror their CSF counterpart and would scarcely be useful for diagnostic and prognostic purposes in neurodegenerative parkinsonism. Conversely, peripheral evaluation of less specific markers such as neurofilament light chain (Nf-L) has demonstrated a high accuracy in distinguishing between PD and PSP when evaluated in serum, especially combined with MRI measurements [38,39]. Nevertheless, o-α-synuclein and p-tau181 might be clinically useful when dosed in other accessible biological matrices that better recapitulate the CNS. As an example, levels of tau aggregates and o-α-synuclein within extracellular vesicles of neural origin showed a very good capability to separate PSP from PD patients, with a modest overlap at the individual level [40]. Additionally, as previously mentioned, synuclein and tau oligomers are highly heterogeneous in molecular weight, structure, and capability to induce misfolding and aggregation [12], but this last feature cannot be detected by common ELISA assays. To overcome this issue, Seeding Amplification Assays (SAAs) have been developed, able to detect the prion-like seeds of synuclein and tau in CSF with high sensitivity and specificity [41,42]. Of note, CSF α-synuclein seeds have been demonstrated to effectively discriminate synucleopathies from tauopathies, particularly when combined with Nf-L and MRI measurements [43,44,45], and it is possible that tau aggregates could be effective as well. If standardized and optimized for easily accessible biological fluids such as plasma or serum, this technique could provide strong support to clinical diagnosis.

In this work, we also identified correlations between serum biomarkers and demographical and clinical variables. We observed a positive association between p-tau181 and age in both HCs and PD patients, which likely reflects the neurodegeneration that physiologically occurs with aging [46,47]. On the other hand, the lack of association with PSP could be due to the limited sample size of this group or a characteristic of this disease. Further studies on larger PSP samples are warranted. Aging is considered the main risk factor for the development of neurodegenerative diseases, including PD and PSP [48]. Such an association has been explained by the fact that most of the phenomena occurring in physiological brain aging, including impaired protein homeostasis, increase in oxidative stress, chronic inflammation, and mitochondrial dysfunction, are also involved in neurodegeneration [49]. Moreover, the histological hallmarks of PD, such as degeneration of dopaminergic neurons, reactive gliosis, and α-synuclein deposits, can be found in healthy elderly subjects, although to a lesser extent [49,50]. Of note, aging can impact levels of α-synuclein misfolding aggregation through the impairment of the ubiquitin–proteosome system, which increases the amount of misfolded proteins within the cell, as well as the levels of oxidation and nitrosylation, which promote aggregation potential [50]. Accordingly, aging has been shown to be a leading factor of α-synuclein oligomerization and propagation in animal models [51,52,53,54]. In humans, lower plasma total α-synuclein levels were associated with aging, while no differences have been reported so far for oligomers [11], in line with our results. PSP is also characterized by late-age onset, with pathological features including neuronal loss, gliosis, and accumulation of misfolded tau protein [55]. Similar to α-synuclein oligomers, tau aggregation and spreading also increase with aging [56,57], while the levels of soluble tau protein decrease [58]. Overall, our and previous findings support the associations between protein levels in body fluids and aging, highlighting the importance of considering age in biomarker studies.

In this work, we also confirmed previous data in CSF on the correlation of disease severity with o-α-synuclein in PD [59,60,61] but not with p-tau181 in PSP [62], which strengthens the notion that serum does not completely recapitulate CSF [59,60]. The association between o-α-synuclein and the severity of PD is interesting, although it contrasts with the low level of this protein in serum, which may reflect its enhanced aggregation in fibrils occurring in these patients, which in turn would leave fewer free molecules to be detected. Further studies in neural exosomes or erythrocytes as well as more investigations using SAAs on serum are needed to better explain this finding. As for the reduction in serum o-α-synuclein levels with age in PSP, such an association is counterintuitive. Considering that tau can induce α-synuclein aggregation [63] and that deposits of both proteins can be found in PSP [64], it is possible that time-mediated accumulation of tau aggregates would promote o-α-synuclein misfolding and deposition, progressively reducing the pool of free molecules of this protein in serum of PSP patients. Further studies to better explain this finding are warranted.

The main strength of our study is that it is the first one investigating serum o-α-synuclein and p-tau181 as discriminating biomarkers for PD and PSP, showing that these two biomarkers are not clinically useful when assessed in this body fluid. Moreover, we matched the three groups for demographic variables to eliminate the possible confounding effects of age and sex. Finally, we measured the selected biomarkers by using commercially available standardized assays, which would allow an easier comparison between studies. Particularly, o-α-synuclein was evaluated by a commercial ELISA kit, while p-tau181 was quantified by a SIMOA, a highly reliable and fully automated technology.

On the other hand, our study has some limitations. First, although diagnoses have been made based on international criteria [1,2] by movement disorder specialists with more than 10 years of experience, pathological confirmation is missing; therefore, some clinical misdiagnoses might have occurred. Second, the number of subjects in each group is relatively small, and further studies on larger patient cohorts are needed to confirm our findings. However, the PSP sample size in the current study is comparable with most studies in the field and reflects the low incidence of the disease. Third, due to the limited number of subjects, patients were not further stratified according to disease severity. Since serum oligomeric α-synuclein showed a positive correlation with motor symptoms in our PD cohort, it is possible that patients with different disease severity would have shown distinct levels of the molecule. On the other hand, the lack of association of p-tau181 suggests that patient stratification would have not shown any difference for this biomarker. Finally, we employed ELISA and SIMOA and focused on o-α-synuclein and p-tau181; further studies comparing different techniques or different α-synuclein and tau forms are warranted.

## 4. Materials and Methods

### 4.1. Subjects

A total of 43 PD patients and 27 PSP patients fulfilling MDS diagnostic criteria [1,2] were recruited at the Movement Disorder Center of the Magna Graecia University of Catanzaro. Of the 27 PSP patients, 21 had probable PSP–Richardson’s Syndrome (PSP-RS), 4 had probable PSP–Parkinsonism (PSP-P), 1 had probable PSP-Frontal (PSP-F), and 1 had probable PSP and Corticobasal Syndrome (PSP-CBS) [2]. Clinical features suggestive of other diseases, MRI signs suggestive of normal pressure hydrocephalus, and normal striatal uptake on 123I-FP-CIT-SPECT were considered as exclusion criteria. Both PD and PSP patients underwent a neurological examination including MDS-UPDRS-III in off-state, and PSP patients were further evaluated by using the PSP Rating Scale [65]. Thirty-nine age- and sex-matched subjects without any neurological or psychiatric disorders were recruited as healthy controls. This study was performed according to the Declaration of Helsinki and approved by the Calabria Region Ethics Committee. All the involved subjects gave written informed consent for participation in the study and the use of their medical records for research purposes.

### 4.2. Serum Collection and Biomarkers Assessment

Serum samples from each subject were collected between 9 a.m. and 12 p.m. in BD Vacutainer^TM^ SST^TM^ Serum Separation Tubes (BD, Franklin Lakes, NJ, USA), centrifuged within 30 min from collection at 3000 rpm for 10 min at 4 °C, aliquoted, and stored at −20 °C until use. For biomarker evaluation, serum aliquots were thawed overnight at 4 °C, mixed thoroughly, and centrifuged at 2200 rpm for 15 min. Each serum aliquot was used once. Free hemoglobin was measured by spectrophotometry to detect hemolysis that could affect o-α-synuclein levels [36]. In all the serum samples, less than 0.1% of hemolysis was detected, below the limit commonly accepted for clinical biochemical tests [66].

Serum o-α-synuclein was evaluated by an ELISA kit (MBS043824, MyBioSource, San Diego, CA, USA) on a Varioskan™ LUX multimode microplate reader (Thermo Fisher Scientific, Waltham, MA, USA). This kit employs mouse monoclonal antibodies against human alpha-synuclein Glu131-Ala140 as capture antibodies, while rabbit polyclonal antibodies against the full-length human oligomeric α-synuclein are used as detection antibodies.

On the other hand, p-tau181 was measured by ultrasensitive single-molecule array (SIMOA) technology (104111, Simoa^®^ pTau-181 Advantage V2.1 Kit, Quanterix, Billerica, MA, USA) on a fully automated Quanterix HD-X™ Automated Immunoassay Analyzer. SIMOA can be considered an evolution of ELISA, allowing the detection of analytes at femtomolar concentrations. In this technology, capture antibodies are attached to paramagnetic beads, while detection antibodies are labeled with β-galactosidase by biotin–streptavidin interactions. Overall, the assay is designed in such a way that a single bead binds a single molecule of an analyte, and each of these immune complexes is labeled by an enzyme. The immune complexes are then loaded on an array of wells whose size can hold a single immune complex. In this way, the fluorescent signal generated by each immune complex is highly concentrated and can be digitally counted, increasing sensitivity to the single-molecule level. The analyte concentration is then calculated as the ratio between the wells containing an immune complex and the total number of wells.

For both the assays, the manufacturers’ instructions were followed. All the measurements were performed in duplicates.

### 4.3. Statistical Analysis

All statistical analyses were performed by using IBM SPSS software version 25 (SPSS Inc., Chicago, IL, USA). Dichotomic variables were compared by the Χ^2^ test. For continuous variables, data were first tested for normality using the Shapiro–Wilk test. Comparisons between three groups were performed by the Kruskal–Wallis test followed by Bonferroni’s correction. Comparisons between two groups were performed by the Mann–Whitney test. Correlations between serum biomarkers and demographical or clinical variables were tested by using Spearman’s test. For all the analyses, *p* < 0.05 was considered significant.

## 5. Conclusions

Although o-α-synuclein and p-tau181 are closely involved in the physiopathology of PD and PSP, respectively, our study suggests that the serum concentrations of these proteins are not useful biomarkers to distinguish patient groups. However, their evaluation in different matrices such as neural-derived extracellular vesicles as well as their assessment with other methodologies such as RT-QuIC, which better recapitulates pathology, may represent valuable strategies for clinical practice.

## Figures and Tables

**Table 1:** Demographic, clinical, and serum features of PSP and PD patients as well as HCs. Data are shown as mean ± SD.

	PSP (n = 27)	PD (n = 43)	HC (n = 39)	*p*-Value
Sex (F/M)	11/16	14/29	17/22	0.57 ^a^
Age at examination (years)	69.1 ± 6.71	69.7 ± 6.80	69.0 ± 6.30	0.88 ^b^
Disease duration (years)	3.5 ± 1.88	5.2 ± 4.54	-	0.28 ^c^
MDS-UPDRS-III	46.5 ± 19.00	28.0 ± 12.57	-	<0.0001 ^c^
PSP Rating Scale	45.5 ± 18.40	-	-	-
HY Staging Scale	3.7 ± 1.24	1.9 ± 0.65	-	<0.0001 ^c^
Serum o-α-synuclein (ng/mL)	8.1 ± 5.99	7.8 ± 6.29	12.3 ± 8.92	0.027 ^b,^*
Serum p-tau181 (pg/mL)	14.6 ± 9.54	15.7 ± 10.30	14.0 ± 7.01	0.97 ^b^
Serum o-α-synuclein/p-tau181 ratio	818.9 ± 173.55	646.7 ± 93.45	984.5 ± 127.59	0.088 ^b^

PSP: progressive supranuclear palsy; PD: Parkinson’s disease; HC: healthy control; MDS-UPDRS-III: MDS—Unified Parkinson’s Disease Rating Scale part III; HY: Hoehn and Yahr; o-α-synuclein: oligomeric α-synuclein; p-tau181: 181Thr-phosphorylated tau. ^a^ Χ^2^-square test. ^b^ Kruskal–Wallis test with Bonferroni’s correction. ^c^ Mann–Whitney test. * *p*-values after Bonferroni’s correction: PSP vs. PD: 1.00; PSP vs. HC: 0.363; PD vs. HC: 0.062.

**Table 2:** Correlation analysis between serum, demographic, and clinical variables in PSP patients.

PSP (n = 27)			Age	Disease Duration	PSP Rating Scale
	o-α-synuclein	Spearman’s rho	−0.51	−0.08	−0.21
		*p*-value	*0.008*	0.71	0.30
	p-tau181	Spearman’s rho	0.04	0.06	0.30
		*p*-value	0.85	0.77	0.14
	o-α-synuclein/p-tau181 ratio	Spearman’s rho	−0.32	0.09	−0.23
		*p*-value	0.11	0.66	0.26

PSP: progressive supranuclear palsy; o-α-synuclein: oligomeric α-synuclein; p-tau181: 181Thr-phosphorylated tau.

**Table 3:** Correlation analysis between serum, demographic, and clinical variables in PD patients.

PD (n = 43)			Age	Disease Duration	MDS- UPDRS-III
	o-α-synuclein	Spearman’s rho	−0.04	0.23	0.43
		*p*-value	0.79	0.14	*0.005*
	p-tau181	Spearman’s rho	0.41	−0.03	0.19
		*p*-value	*0.006*	0.84	0.23
	o-α-synuclein/p-tau181 ratio	Spearman’s rho	−0.33	0.10	0.11
		*p*-value	*0.035*	0.54	0.48

PD: Parkinson’s disease: MDS-UPDRS-III: MDS—Unified Parkinson’s Disease Rating Scale part III; o-α-synuclein: oligomeric α-synuclein; p-tau181: 181Thr-phosphorylated tau.

**Table 4:** Correlation analysis between serum biomarkers and age in HC.

HC (n = 39)			Age
	o-α-synuclein	Spearman’s rho	0.31
		*p*-value	0.06
	p-tau181	Spearman’s rho	0.47
		*p*-value	*0.002*
	o-α-synuclein/p-tau181ratio	Spearman’s rho	0.12
		*p*-value	0.49

HC: healthy control; o-α-synuclein: oligomeric α-synuclein; p-tau181: 181Thr-phosphorylated tau.

## Data Availability

Due to privacy restrictions, the data supporting the results of this study are not publicly available and are available from the corresponding author upon reasonable request.
